# Anti-Müllerian hormone independently affect mtDNA copy number in human granulosa cells

**DOI:** 10.1186/s13048-022-01047-4

**Published:** 2022-10-13

**Authors:** Anom Bowolaksono, Ayu Mulia Sundari, Muhammad Fauzi, Mila Maidarti, Budi Wiweko, Kresna Mutia, Pritta Ameilia Iffanolida, Ririn Rahmala Febri, Astari Dwiranti, Hiroaki Funahashi

**Affiliations:** 1 Cellular and Molecular Mechanisms in Biological System (CEMBIOS) Research Group, Department of Biology, Faculty of Mathematics and Natural Sciences, Universitas Indonesia, Kampus FMIPA UI, 16424 Depok, Indonesia; 2 Indonesian Reproductive Science Institute (IRSI) Research and Training Center, Jakarta, Indonesia; 3 Department of Diabetes, Endocrinology, and Clinical Nutrition, Graduates School of Medicine, Kyoto University, Kyoto, Japan; 4 Human Reproductive, Infertility, and Family Planning (HRIFP) Research Center, Indonesian Medical Education and Research Institutes, Faculty of Medicine, Universitas Indonesia, Jakarta, Indonesia; 5 Department of Obstetrics and Gynecology, Division of Reproductive Endocrinology and Infertility, Faculty of Medicine, Universitas Indonesia, Jakarta, Indonesia; 6 Graduate School of Natural Science and Technology, Okayama University, Tsushima-Naka, Kita-ku, 700-8530 Okayama, Japan

**Keywords:** Anti-Müllerian hormone, Mitochondrial DNA (mtDNA), Aging, Granulosa cells, Fertility

## Abstract

**Background:**

Recently, as a delayed childbearing trend is emerging in modern women’s adulthood, diminished reproductive potential due to age-related changes is more prevalent. Reduction in the abundance of mitochondrial DNA (mtDNA) copies and circulating anti-Müllerian hormone (AMH) have been separately reported with aging, contributing to the decrease in successful reproduction. However, there are limited reports on the impact of age on mtDNA and AMH in the same individual and whether mtDNA copy numbers are influenced by age and AMH.

**Methods:**

In the present study, we utilized a real-time quantitative PCR (RT-qPCR) to quantify the mtDNA copy number of granulosa cells obtained from 43 women undergoing an in vitro fertilization (IVF)/intracytoplasmic sperm injection (ICSI) program.

**Results:**

According to our analysis, a significant correlation was observed between age and mtDNA copy number (r = −0.54, P < 0.001) and between age and AMH level (r = −0.48, P < 0.001) of the same individual. There was also a positive correlation between mtDNA copy number and AMH (r = 0.88, P < 0.001) with AMH level falling as mtDNA decreases. In our regression, age and AMH were shown to have low collinearity (VIF = 1.297) but only AMH was correlated with mtDNA quantity (P < 0.001).

**Conclusion:**

Our study suggests that both mtDNA and AMH abundance are influenced by age and that AMH levels independently affect mtDNA copy number regardless of age. Further research is required to understand the role of AMH on mitochondria bioenergetics.

## Introduction

In contrast to the male physiology where gametes are newly produced in the testes thus fertility maintained over a lifetime, women are born with a finite germinal reserve [1, 2]. The follicular assembly, a developmental process by which individual oocytes assemble into primordial follicles, begins in the mid-gestation when a few thousand primordial germ cells populate the genital ridges and massively proliferate resulting in 7 × 10^6^ oogonia [1, 3]. A counterbalance mechanism of programmed cell death is subsequently put in motion following this transition, reducing 85% of the potential oocyte population, leaving only a million primordial follicles at birth and only 400,000 oocytes when women enter puberty [4–6]. The squamous granulosa is concomitantly developed into a cuboidal, proliferates, and the follicle sequesters theca from the adjacent stroma [7]. This transition is physiologically non-reversible and that follicle population represents a female’s total reproductive potential. Consequently, aging is a critical determinant of women’s reproductive wellbeing.

Depletion of mitochondrial DNA (mtDNA), a small circular chromosome located in mitochondria, has emerged as a critical factor in women’s aging, with decreases of 0.4 copies of mtDNA per year [8, 9]. Encoding respiratory complex subunits required for energy cellular supplies, mtDNA has been shown to contribute to oocyte maturation and embryonic development [10]. As such, it was reported that mtDNA and ATP content increased during bovine oocyte maturation, meanwhile in vitro maturation (IVM) on porcine oocytes demonstrated that mtDNA copy numbers increased from GV to MII stage during the treatment [11, 12]. On the other hand, it has been shown that disturbances in energy supply due to mitochondrial dysfunction are responsible for adverse outcomes, including maturation failure, fertilization failure, and abnormal chromosomal segregation, strengthening the significant role of mtDNA on successful reproduction [13, 14].

In addition to deterioration in mtDNA, a decline in the anti-Müllerian hormone (AMH), a hormone produced by granulosa cells of growing follicles, is another major consequence of aging in women. Studies reported that circulating AMH levels consistently decline with aging and is accelerated between 45 and 50 years of age [15, 16]. In the ovary, AMH plays an important role in the regulation of ovarian follicle growth by inhibiting initial follicle recruitment and decreasing the dependent growth of small antral follicles to the follicle-stimulating hormone (FSH) [17]. With regard to its effect on diminished fertility fitness, AMH level is strongly correlated with antral follicle count (AFC), which, therefore, represents the number of remaining follicles in the ovaries [18, 19]. Further evidence shows that AMH is a promising marker of the late menopausal transition and ovarian reserve, better than chronological age, AFC, or ovarian volume [20, 21]. In women undergoing in vitro fertilization (IVF), unpleasant results including a high rate of cancellation and low clinical pregnancy rate were observed in low-AMH subjects [22, 23].

To date, although the effect of chronological age on mtDNA and AMH has widely been separately described in animals and humans, there is still limited evidence regarding the effect of age on mtDNA and AMH in the same individual. Additionally, no studies have investigated how age and AMH affect mtDNA copy number, and whether these effects are independent of each other. Therefore, we aim to scrutinize the effect of age on mtDNA copy number and AMH level, concomitantly exploring the possible effect of age and AMH on mtDNA abundance.

## Materials and methods

### Patients and sample collection

A total of 43 women undergoing IVF with intracytoplasmic sperm injection (ICSI) were recruited from Yasmin Infertility Clinic, Dr. Cipto Mangunkusumo General Hospital, Jakarta, Indonesia according to inclusion criteria: women undergoing first or subsequent cycles, had primary or secondary infertility and signed informed consent. Meanwhile, subjects who were diagnosed with PCOS according to the Rotterdam criteria, identified as poor responders, had endometriosis, or had incomplete data records were excluded from the study. For body composition grouping, BMI was classified as previously described [24].

For sample collection, granulosa cells (GCs) were collected from the successfully retrieved cumulus-oocyte complex (COC) at the time of ovum pick up (OPU). After aspiration, COCs were rinsed in a buffer medium (GMOPS™ PLUS, Vitrolife, Sweden) and incubated in a humidified incubator at 37 °C under 6% CO2 for 3 h in a culture medium. Following incubation, COCs were immersed in hyaluronidase to remove cell cumulus. Samples from each subject were subsequently subjected to the proceeding treatment to separate GCs from undesirable cells. Briefly, the sample was pooled into a 14 ml sterile container and was centrifuged at 160×G for 10 min. Subsequently, the supernatant was discarded and the pellet was resuspended in phosphate buffer saline (PBS) to 6 mL of volume. The mixture was re-centrifuged at 160×G for 10 min and this process was repeated two further times.

Following the last centrifugation, the homogenate was transferred into a 14 mL tube containing 2 mL of Ficoll-Paque with a 1:3 ratio of homogenate to Ficoll-Paque. The mixture was re-centrifuged at 155×G for 35 min resulting in four distinct layers. GCs that formed a clear ring layer in the second layer from the top were pipetted, resuspended in a buffer medium to a final volume of 6 mL, and centrifuged at 155×G for 10 min. Finally, the supernatant was resuspended in a 1 mL buffer medium and stored in a 1.5 mL tube for the subsequent experiment.

### DNA extraction

The total DNA was extracted using a Geneaid™ DNA isolation kit (Geneaid, Taipei) according to the manufacturer’s protocol. Briefly, 900 µL of RBC lysis buffer was transferred into 300 µL of samples in a 1.5 ml tube. Following 5 min of incubation at room temperature, the mixture was centrifuged at 3,000×G for 5 min and the supernatant was discarded. To initiate cell lysis, 300 µL of cell lysis buffer was added to the resulting pellet, mixed by a vortex, and incubated at 60 ℃﻿ for 10 min to yield a homogenous suspension. To further eliminate protein residues, 100 µL of protein removal buffer was subsequently added to the sample lysate followed by centrifugation at 16,000×G for 3 min. The resulting supernatant was subsequently transferred to a clean 1.5 mL tube and 600 µL of isopropanol was added. The sample was centrifugated at 16,000×G for 5 min. Immediately after centrifugation, the supernatant was carefully discarded and 300 µL of 70% ethanol was added to wash the pellet. Centrifugation at 16,000×G for 3 min was then performed and 100 µL of DNA Hydration Buffer was added. DNA concentration was determined by reading absorbance at 260 nm.

### Real-time quantitative PCR (RT-qPCR)

RT-qPCR was performed on the Techne PrimePro 48 (Techne, Germany) in an Eco 48-well plate (Cole Parmer, UK) using SensiFAST SYBR® No-ROX Kit (Bioline, Meridian, USA). For each individual assay, 15 µL reaction mix containing 1X SensiFAST SYBR® No-ROX Mix, 400 nM forward primer, 400 nM reverse primer, and 25 ng template sample were loaded into one well. mtDNA copy number was quantified by the ratio of a target mitochondrial gene to a reference of the nuclear gene (MT-CO1/B2M) using the selected primers: MT-CO1 (Forward: 5’− CTT CGT CTG ATC CGT CCT AAT C − 3’; Reverse: 5’ − TTG AGG TTG CGG TCT GTT AG − 3’) and B2M (Forward: 5’ − CCC ACT GTC ACA TGC ATT ACT − 3’; Reverse: 5’ − GGT TGA GTT GGA CCC GAT AAA − 3’), respectively. Amplification was carried out in 40 cycles according to the thermal profile as follows: polymerase activation at 95 ℃ for 10 min, denaturation at 95 ℃ for 45 s, annealing at 54 ℃ for 1 min, and extension at 72 ℃ for 45 s. All experiments were performed in duplicate.

### Data analysis

Subjects’ characteristics were presented as number and percentage, and mean ± standard deviation or median (minimum-maximum) if applicable. The D’Agostino & Pearson normality test was used to determine the distribution of subject characteristics. Data transformation with square root function was performed as necessary. For univariate analysis, differences between categories of subjects were assessed by ANOVA and t-test, while Pearson’s correlation with Bonferroni correction was used to demonstrate correlation among continuous variables. Multivariate analysis was conducted using the least-square method. All statistical analysis was performed using R version 4.2.

## Result

**Fig. 1:**
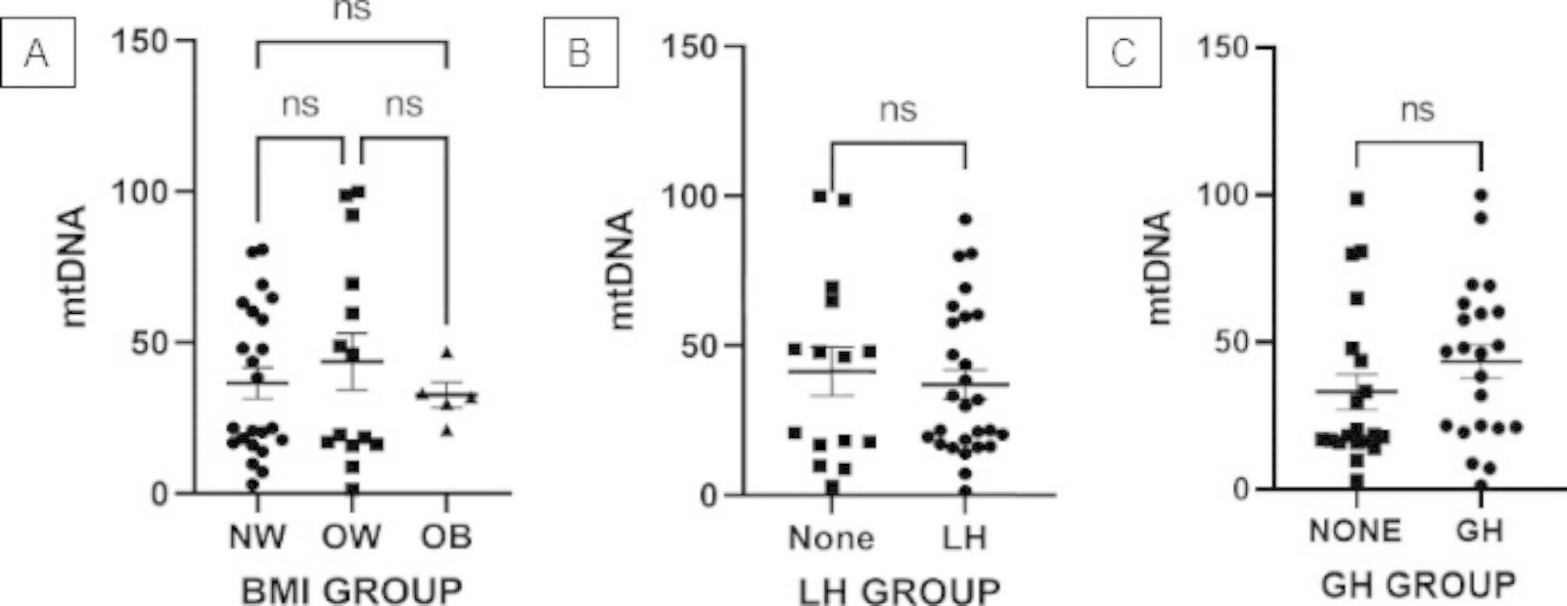
Comparison of mitochondrial DNA (mtDNA) copy number between normal weight (NW) [N = 24], overweight (OW) [N = 14], and obese (OB) [N = 5] subjects (A), between LH-administered [N = 27] and non-administered subjects [N = 16] (B), and between GH-administered [N = 22] and non-administered subjects [N = 21] (C). mtDNA copies are relatively quantified to the amount of B2M. All values are expressed as mean ± standard deviation (SD) obtained from two separate experiments (Duplo). (BMI group was analyzed by ANOVA; LH and GH group were analyzed by t-test). *ns: not significant

**Fig. 2:**
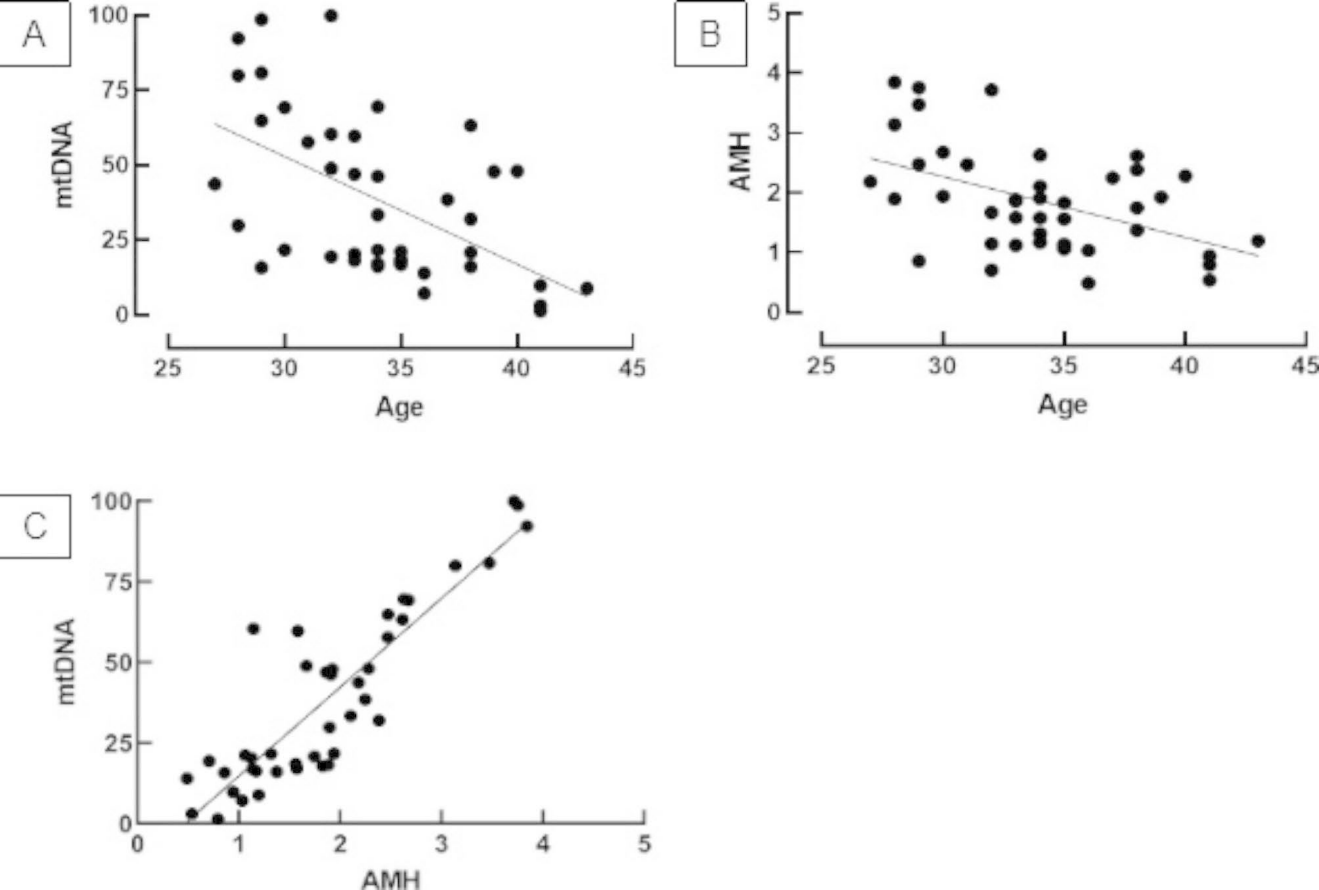
Simple linear regression between age and mtDNA copy number (r = −0.54, P < 0.001) (A), between age and AMH level level (r = −0.48, P < 0.001) (B), and between AMH level and mtDNA copy number (r = 0.88, P < 0.001) (C). mtDNA copies are relatively quantified to the amount of B2M. All values are expressed as mean ± standard deviation (SD) obtained from two separate experiments (Duplo) (Data was analyzed using Pearson’s correlation)

**Fig. 3:**
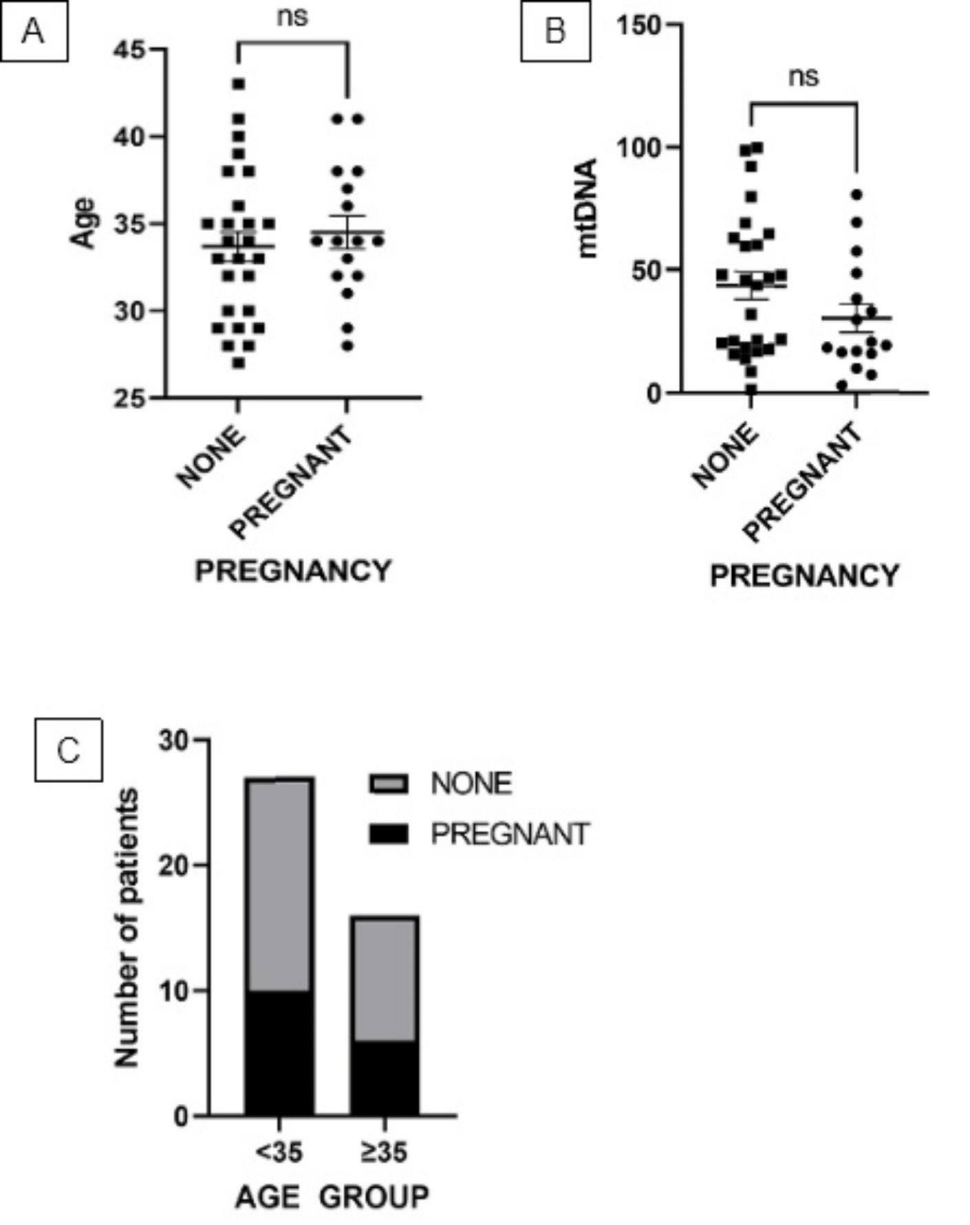
Comparison of pregnancy frequency according to age (A), mtDNA copy number (B), and age (C) when stratified into < 35 and ≥ 35 years old. mtDNA copies are relatively quantified to the amount of B2M. All values are expressed as mean ± standard deviation (SD) obtained from two separate experiments (Duplo) (Pregnancy data according to mtDNA copies and age were analyzed using a t-test; pregnancy proportion according to age group was analyzed using Fisher’s exact test). *ns: not significant

A total of 43 participants were enrolled in this study and baseline characteristics are summarized in Table 1. To ascertain the possible effect of energy metabolism and adjuvant therapy on mtDNA copy number, subjects were categorized by body mass index (BMI) and administration of Luteinizing Hormone (LH) and Growth Hormone (GH), and groups were compared using ANOVA and t-tests (Fig. 1 A-C). After grouping, the distribution of normal weight (NW), overweight (OW), and obese subjects were 24, 14, and 5 while the proportion between the recipient and non-recipient of LH and GH was 27 vs. 16 and 22 vs.21, respectively. mtDNA levels in normal weight, overweight, and the obese group were comparable (Fig. 1 A), and neither LH nor GH administration had an effect on mtDNA levels (Fig. 1B C, respectively). Meanwhile, correlation analysis showed a meaningful correlation between age and mtDNA copy number (r = −0.54, P < 0.005; Fig. 2 A) and between age and AMH level (r = −0.48, P < 0.05; Fig. 2B). Interestingly, there is a significant positive correlation between mtDNA copy number and AMH level (r = 0.88, P < 0.001; Fig. 2 C) as AMH level declines with mtDNA depletion. Multivariate analysis further demonstrated that both age and AMH exert an effect on mtDNA copy number independently of each other (VIF = 1.297; Table 2), but only AMH level was correlated with mtDNA (P < 0.001; Table 2). In terms of primary outcomes, there is no association either between age and pregnancy (P = 0.54, Fig. 3 A) or between mtDNA and pregnancy (P = 0.15, Fig. 3B) although a decreased tendency of pregnancy with age was observed when subjects were stratified into < 35 and ≥ 35 years of age (64% vs. 60%) (Fig. 3 C).

**Table 1:** Demographic and baseline characteristics of participants

Characteristics	Mean ± SD Median (Min-Max) n (%)
Age (years)	32.16 ± 4.14
Type of infertility [n (%)]	
Primary	39 (90.70)
Secondary	4 (9.30)
BMI (kg/m^2^)	24.67 ± 3.80
Etiology of infertility [n (%)]	
Tubal factor	12 (27.90)
Male factor	13 (30.24)
Unexplained	18 (41.86)
AMH (ng/mL)	3.35 (0.02 – 14.77)
**Stimulation**	
Total gonadotropin dosage (IU)	3181 (900 – 5325)
Endometrial thickness (mm)	9.73 ± 3.99
Number of oocytes retrieved	13.42 ± 7.09
Number of mature oocytes	12.05 ± 5.92
**Embryo characteristics**	
Number of fertilized oocytes	8.12 ± 6.40
Number of transferred embryo	0 (0 – 2)

**Table 2:** Result of multivariate linear regression analysis

Variable	β	SE	|t|	P-Value	P-Value Summary	VIF	R^2^
Age	-1.043	0.5448	1.915	0.0628	ns	1.297	0.80
AMH	25.19	2.785	9.801	< 0.0001	****		

## Discussion

Age has been known to affect fertility in women, with aging resulting in diminished reproductive capacity due to progressive atresia and exhaustion of oocytes [25]. According to previous reports, it has been observed that aging in the reproductive system is significantly faster compared to other regions. Accumulation of oxidative damage due to ovarian aging concomitantly causes follicle number drop and oocyte quality decay leading to gradual falls in female fertility [26]. In the present study, we demonstrated a decline in mtDNA copy number and AMH level with age in the same individual, and for the first time observed the independent effect of AMH level on the decrease of mtDNA copy number.

As mtDNA is more vulnerable than nuclear DNA (nDNA), we evaluated the effect of BMI and adjuvant hormone therapy on mtDNA copy number to eliminate the possible bias of mtDNA changes due to differences in energy metabolism and type of medication. According to our analysis, mtDNA copy number is comparable in all BMI groups, contradicting the findings of other studies. Bordoni et al. (2019) reported a negative correlation between mtDNA copy number and BMI in female subjects, while Skuratovskaia et al. (2018) demonstrated that BMI values are positively associated with mtDNA abundance [27, 28]. However, these results were reported from tissues other than GCs, with different patterns of mtDNA copies being reported in different tissues. A positive link between BMI and mtDNA copy number was observed in subcutaneous adipose tissues and vascular adipose tissue, but it was conversely a negative correlation in peripheral blood [28]. Interestingly, the abundance of mtDNA was also varied in different areas of the same tissue demonstrating that mtDNA replication is tissue-specific [28]. Accordingly, the expression of mtDNA in GCs appears to not be affected by body composition resulting in a constant count in this study. In addition, it emerges that the link between mtDNA and BMI was more frequently observed in individuals with diseases [29, 30]. Considering participants involved in this study were generally healthy, we assumed the likelihood of the opposite result was prominent.

Due to the adverse influence of aging, an “add-on” or adjuvant medical intervention is commonly given, either alone or in combination, to the standard protocol of IVF to improve outcomes [31, 32]. LH and GH are two substances ubiquitously utilized for this purpose. Involved in the completion of folliculogenesis and ovulation induction, LH supplementation during stimulation was reported to significantly increase the implantation rate of older women [33]. GH co-treatment patients were also reported to have a higher pregnancy rate compared to the non–GH-treated patients of advanced age [34]. In the present study, the mtDNA copy number was shown to be unaffected by the presence of both LH and GH. Notably, an endocrine disturbance due to an increase in the serum FSH followed by a decline in the basal androgen and the impairment of androstenedione synthesis has been described as a major consequence of ovarian aging in women [35–37]. An increase in aromatase activity in the early follicular phase is most likely due to the higher FSH levels in older women. Unfortunately, our study did not determine whether the level of basal FSH serum was upregulated at the start of ovarian stimulation since the FSH level was not measured. The basal high FSH levels can be valuable to forecast the ovarian response to stimulation in poor responder patients. The high FSH levels can be caused by the decline of granulosa cell numbers leading to lower inhibin expression, as proven by low AMH levels [38].

LH is reported to restore follicular milieu in the ovary via androgen production required for aromatization, thus recovering oocyte quality [33]. On the other hand, an evaluation in older subjects showed that GH co-treatment has a reversal effect on the down-regulation of FSHR, BMPR1B, and LHR density of the granulosa cells [34]. It is apparent from our observations that improved fertility outcomes due to LH and GH supplementation are not mediated by mtDNA, and so should not have an effect on the findings of this study.

Similar to previous reports, our study confirmed a significant correlation between aging and mtDNA copy depletion, and also between aging and AMH level decrease. Consistent with our finding, it has been estimated that human mtDNA copies decrease by about 4 copies every 10 years, which is responsible for impaired post-implantation development and increased risk of subfertility [8, 39, 40]. Additionally, an inverse relationship between AMH and age has also been noted, where a significant decline from 6.71 ng/mL to 0.68 ng/mL and a decrease from 0.27 to 0.12 ng/mL/year was observed among subjects aged 17 – 20 years to 51 – 61 years and between subject aged 35 and over 35 [41]. There is solid evidence that one important contribution of aging is the accumulation of unrepaired DNA damage, in which a higher rate of mutation, oxidative damages, and lower replication fidelity was common to mtDNA compared to the nDNA, an inherits maternally and paternally genetic material containing information of the cell [42–44]. As AMH is secreted by granulosa cells of growing follicles, an increase in age consequently results in a gradual reduction of AMH level, which was reported to peak at puberty and become undetectable during menopause [15, 45]. Accordingly, the accumulation of DNA damage and endocrine changes are thought to be the underlying mechanism of our findings.

In the present study, we demonstrated that age and AMH are significantly correlated with mtDNA copy number, and that AMH is strongly associated with mtDNA copy number independent of age. To the best of our knowledge, this is the first report showing the association of AMH and mtDNA in the same individual, and therefore there is no corresponding available report. Nevertheless, it is known that one of the detrimental consequences of mitochondrial damage experienced by all cells including GCs is an increased production of reactive oxygen species (ROS) which disrupt the mitochondrial respiratory chain. Following the complex I blockade, the electron transport chain upregulates ROS production due to an elevated reduction of oxygen into superoxide, resulting in an inactivation of the iron-sulfur cluster, the terminal step of electron transfer of complex I. These ROS subsequently damage all biological macromolecules, including proteins, lipids, and DNA [46, 47]. Interestingly, supplementation of selenium and vitamin E have been reported to significantly improve AMH levels and AFC in occult premature ovarian insufficiency in women [48]. According to the abovementioned report, it seems that antioxidants decrease ROS levels and improve the respiration chain. Therefore, the possible explanation for our finding is that this causative cascade is bidirectional, thus lower AMH levels reflect the depletion in mtDNA abundance.

## Conclusion

The present study reveals that age is inversely correlated with mtDNA copy number and circulating AMH level, as shown elsewhere. Moreover, we found that age and AMH are associated with mtDNA abundance and that AMH level is strongly correlated with mtDNA copy number, independent of age. Future studies are required to understand the role of AMH on mitochondria.

## Data Availability

The data that support the findings of this study are available from the corresponding author upon reasonable request.

## Abbreviations

AFC: antral follicle count.
AMH: anti-Müllerian hormone.
BMI: body mass index.
COC: cumulus-oocyte complex.
FSH: follicle-stimulating hormone.
GC: granulosa cell.
GH: growth hormone.
ICSI: intracytoplasmic sperm injection.
IVF: in vitro fertilization.
IVM: in vitro maturation.
LH: luteinizing hormone.
mtDNA: mitochondrial DNA.
nDNA: nuclear DNA.
OPU: ovum pick up.
PBS: phosphate buffer saline.
ROS: reactive oxygen species.
RT-qPCR: real-time quantitative PCR.
SD: standard deviation.
